# Swelling/Floating Capability and Drug Release Characterizations of Gastroretentive Drug Delivery System Based on a Combination of Hydroxyethyl Cellulose and Sodium Carboxymethyl Cellulose

**DOI:** 10.1371/journal.pone.0116914

**Published:** 2015-01-24

**Authors:** Ying-Chen Chen, Hsiu-O Ho, Der-Zen Liu, Wen-Shian Siow, Ming-Thau Sheu

**Affiliations:** 1 School of Pharmacy, College of Pharmacy, Taipei Medical University, Taipei, Taiwan; 2 Graduate Institute of Biomedical Materials and Engineering, Taipei Medical University, Taipei, Taiwan; 3 Center for General Education, Hsuan Chuang University, Hsinchu, Taiwan; 4 Cancer and Chinese Herb Research Center, Taipei Medical University Hospital, Taipei, Taiwan; University of California, Merced, UNITED STATES

## Abstract

The aim of this study was to characterize the swelling and floating behaviors of gastroretentive drug delivery system (GRDDS) composed of hydroxyethyl cellulose (HEC) and sodium carboxymethyl cellulose (NaCMC) and to optimize HEC/NaCMC GRDDS to incorporate three model drugs with different solubilities (metformin, ciprofloxacin, and esomeprazole). Various ratios of NaCMC to HEC were formulated, and their swelling and floating behaviors were characterized. Influences of media containing various NaCl concentrations on the swelling and floating behaviors and drug solubility were also characterized. Finally, release profiles of the three model drugs from GRDDS formulation (F1-4) and formulation (F1-1) were examined. Results demonstrated when the GRDDS tablets were tested in simulated gastric solution, the degree of swelling at 6 h was decreased for each formulation that contained NaCMC in comparison to those in de-ionized water (DIW). Of note, floating duration was enhanced when in simulated gastric solution compared to DIW. Further, the hydration of tablets was found to be retarded as the NaCl concentration in the medium increased resulting in smaller gel layers and swelling sizes. Dissolution profiles of the three model drugs in media containing various concentrations of NaCl showed that the addition of NaCl to the media affected the solubility of the drugs, and also their gelling behaviors, resulting in different mechanisms for controlling a drug’s release. The release mechanism of the freely water-soluble drug, metformin, was mainly diffusion-controlled, while those of the water-soluble drug, ciprofloxacin, and the slightly water-soluble drug, esomeprazole, were mainly anomalous diffusion. Overall results showed that the developed GRDDS composed of HEC 250HHX and NaCMC of 450 cps possessed proper swelling extents and desired floating periods with sustained-release characteristics.

## Introduction

Among administration routes utilized for drug delivery, the oral route remains the preferred way because of low costs, ease of administration, and better patient compliance. However, the conventional immediate-release oral dosage form has a problem of a transient overdose followed by a long period of underdosing, which can be overcome by designing controlled drug delivery systems. The current oral controlled release technology enables the release of many drugs at a constant rate for a period of 12~24 h. However, it is not beneficial for drugs that are only absorbed in the stomach or in the upper small intestine, exhibit local efficacy in the stomach, are unstable, or have low solubility in high-pH environments [1,2]. Hence, the development of gastroretentive drug delivery system (GRDDS) needs to be promoted.

Other than being able to continuously release drugs in the absorption site of the stomach, GRDDS provides a more effective treatment of local stomach disorders and achieves a greater and more prolonged therapeutic efficacy, thus reducing the frequency of administration [3,4]. So far, various approaches such as floating [5–12], bioadhesive [13], and swelling/expanding [14–20] systems have been used to increase gastric retention times, enhance drug absorption within the stomach, and promote a controlled release rate of active substances in the gastrointestinal (GI) tract [3,4,21–23]. In particular, swelling/expanding GRDDS is highlighted by many granted patents for the optimization of polymer ratios. Combining different gastroretentive mechanisms is studied to increase gastroretentive capabilities. Tablets blending hydroxypropyl methylcellulose (HPMC) and Carbopol [24], and those containing sodium carboxymethyl cellulose (NaCMC), hydroxypropyl cellulose (HPC), and carbonate [25] showed good floating and bioadhesive properties. Effervescent tablets of ciprofloxacin were made using NaCMC, HPMC, polyacrylic acid, polymetacrylic acid (MAA), citric acid, and sodium bicarbonate [26]. Moreover, Varshosaz et al. [27] also developed swellable, floating, and sustained-release tablets of ciprofloxacin using a combination of a hydrophilic polymer (HPMC), swelling agents (crospovidone, sodium starch glycolate, and croscarmelose sodium) and an effervescent substance (sodium bicarbonate). Adding sodium bicarbonate to the gel matrix-forming tablets increases the hydration volume and buoyancy [28]. Metformin was formulated as a floating matrix tablet using a gas-generating agent (sodium carbonate) and a gel-forming hydrophilic polymer (HPMC), and demonstrated a floating time of more than 8 h [29]. A new GRDDS of ofloxacin was developed using floating (sodium bicarbonate), swellable (crospovidone and beta-cyclodextrin), and bioadhesive materials (psyllium husk and HPMC) [30]. Kollidon SR (with a low density) and sodium bicarbonate were used to compensate for the deficient floating properties of propranolol in the preparations of directly compressible GRDDS [31,32]. Swellable and floating tablets that used HPMC, swelling agents (crospovidone, sodium starch glycolate, and croscarmellose sodium), and a gas-generating substance (sodium bicarbonate) were found to show better gastroretentive abilities and sustained drug release than those of CIFRAN OD, of which the main mechanism of gastroretention worked by way of the swellability of hydrophilic polymer [27].

In a preliminary study, hydrocolloid tablets made of PEO 8000K were found to have the largest swelling index, followed by those made of hydroxyethyl cellulose (HEC) and NaCMC. However, the floating ability of these polymeric materials was in the following order: HEC > NaCMC > PEO 8000K. HEC is a nonionic and water-soluble polymer widely used in pharmaceutical formulations [33,34]. As expected, swelling to a larger size and floating in gastric fluids can further extend stomach retention; thus, it was thought that simply incorporating negatively charged NaCMC in the HEC matrix might expand the gel matrix of HEC via repulsive forces to a greater extent to enhance its gastroretentive ability.

Ciprofloxacin, a broad-spectrum antibiotic with 70% bioavailability, is mainly absorbed in the upper GI tract, up to the jejunum. Hence, the drug was suggested for design to deliver substances to the stomach, the so-called absorption window, by increasing gastric residence times of the dosage forms. Different sustained-release formulations for ciprofloxacin, Cipro XR or Cipro XL, was reported which rapidly achieved therapeutic drug levels and which was maintained over the course of 24 h, allowing once-daily dosing. It provides higher maximum plasma concentrations (C_max_) with lower interpatient variability than the conventional, immediate-release, twice-daily formulation, and thus decreased patient noncompliance, the risk of treatment failure, and the spread of antibiotic resistance [26]. Metformin is also better absorbed in the upper intestine [29]. There are three advantages of GRDDS to deliver esomeprazole: (1) The target site of esomeprazole is parietal cells in the stomach. The longer the residence time in the stomach is prolonged, the more effective acid inhibition is. (2) GRDDS maintains a certain concentration by gradually releasing esomeprazole, therefore the proton pump is persistently inhibited during a 24-hour period. (3) Esomeprazole given through divided doses is efficient; however, this regimen is not convenient for patients. Reducing the administration frequency improves patient compliance [35,36]. Overall, metformin, ciprofloxacin, and esomeprazole were suggested to be candidates for incorporation into GRDDS. Therefore, the aim of this study was to characterize the behaviors of swelling and floating of GRDDS composed of HEC and NaCMC and to optimize HEC/NaCMC GRDDS for incorporation of these three model drugs (metformin, ciprofloxacin, and esomeprazole) with different solubilities.

## Materials and Methods

### Materials

Natrosol hydroxyethylcellulose 250HHX Pharma (HEC 250 HHX) and Aqualon sodium carboxymethylcellulose (NaCMC, with respective viscosities of 450 and 2500 cps) were supplied by Hercules (Düsseldorf, Germany). Poly(ethylene oxide) with molecular weight 8 x 10^6^ (PEO 8000K) was obtained from the Dow Chemical (Midland, Michigan, USA). Metformin HCl, ciprofloxacin HCl, and esomeprazole sodium were respectively purchased from Flame Pharmaceuticals (Andhra Pradesh, India), Neuland Laboratories (Andhra Pradesh, India), and Glenmark Generics (Bharuch Gujarat, India). All other chemical substances or excipients were reagent or pharmaceutical grade.

### Evaluation of formulations

**(1) Preparation of tablets**. All excipients (HEC 250HHX, PEO 8000K, and NaCMC with viscosity of 450 and 2500 cps) were separately passed through a no. 60 sieve. Polymers were then mixed according to the weight proportions listed in Table 1. The mixture was then compressed on a Carver Laboratory Press tableting machine using 1-ton flat-faced punches (diameter 8 mm) for 6 s. The code F1–4 means there were 40% NaCMC of 450 cps and 60% HEC 250HHX used; F2–4 means there were 40% NaCMC of 2500 cps and 60% HEC 250HHX applied; while G1–4 means 40% NaCMC of 450 cps and 60% PEO 8000K were used.

**Table 1:** Formulation compositions of the swellable/floatable gastroretentive drug delivery system and their swelling ability and floating behavior in deionized water and simulated gastric solution.

Rx	Components (%)				Observation		
	HEC 250HHX	PEO 8000K	NaCMC		Swelling index (*S*_w_)		Float / Sink
			450cps	2500cps	2 h	6 h	
F1–0	100	-	0	-	3.76±0.01	6.99±0.10	Float (>4h)
F1–1	90	-	10	-	4.57±0.07	8.40±0.42	Float (>2h)
F1–2	80	-	20	-	4.90±0.38	10.63±0.33	Float (>4h)
F1–3	70	-	30	-	5.75±0.24	12.61±0.01	Float (>2h)
F1–4	60	-	40	-	5.91±0.32	13.45±0.58	Float (>4h)
F1–5	50	-	50	-	5.80±0.16	13.95±0.34	Sink
F1–6	40	-	60	-	6.20±0.06	13.32±0.14	Sink
F1–7	25	-	75	-	6.19±0.06	9.95±0.29	Float (>4h)
F1–8	10	-	90	-	5.57±0.37	7.19±0.44	Sink
F1–9	0	-	100	-	4.83±0.45	2.28±0.04	Float (>2h)
F1–0*	100	-	0	-	4.05±0.68	7.54±1.17	Float (>8h)
F1–1*	90	-	10	-	3.99±0.25	7.07±0.40	Float (>8h)
F1–2*	80	-	20	-	3.68±0.06	6.61±0.37	Float (>8h)
F1–3*	70	-	30	-	3.60±0.15	6.33±0.02	Float (>8h)
F1–4*	60	-	40	-	3.41±0.07	6.38±0.17	Float (>8h)
F1–5*	50	-	50	-	3.35±0.16	5.81±0.21	Float (<6h)
F1–6*	40	-	60	-	3.22±0.22	4.98±0.15	Float (<4h)
F1–7*	25	-	75	-	2.81±0.11	4.00±0.13	Float (<4h)
F1–8*	10	-	90	-	Erosion	Erosion	Float (<2h)
F1–9*	0	-	100	-	Erosion	Erosion	Float (<1h)
F2–1	90	-	-	10	5.11±0.47	10.40±0.18	Float (>4h)
F2–2	80	-	-	20	6.82±0.25	13.48±0.62	Sink
F2–3	70	-	-	30	8.72±0.11	16.45±1.82	Float
F2–4	60	-	-	40	8.18±0.45	12.66±2.66	Float
F2–5	50	-	-	50	7.86±0.40	13.24±0.40	Sink
F2–6	40	-	-	60	9.79±0.08	10.09±0.02	Sink
F2–7	25	-	-	75	7.32±0.23	7.39±0.03	Float (>4h)
F2–8	10	-	-	90	7.27±0.26	4.71±0.28	Sink
F2–9	0	-	-	100	6.38±1.38	0.68±0.41	Float (>2h)
G-0		100	0	-	4.30±0.50	8.67±0.42	Sink
G-1	-	90	10	-	4.61±0.09	9.96±0.84	Sink
G-2	-	80	20	-	4.83±0.12	11.01±0.06	Sink
G-3	-	70	30	-	5.51±0.14	11.44±0.10	Sink
G-4	-	60	40	-	5.63±0.23	12.30±0.30	Sink

*: Experiments were conducted in simulated gastric solution. For the rest, experiments were conducted in deionized water.

**(2) Determination of the swelling index**. Swelling studies were conducted using the Vankel Dissolution Apparatus (VK7020S, Varian, Palo Alto, CA, USA). No rotation speeds were applied. Pre-weighted tablets were immersed in 500 mL of the medium (deionized water, DIW; simulated gastric solution, 0.1N HCl) and maintained for 8 h at 37.0 ± 0.5°C. At predetermined time intervals (0, 0.5, 2, 4, 6, and 8 h), the swollen tablets were removed from the solution, immediately wiped with a paper towel to remove surface droplets, and weighed. The swelling index (*S*_w_) was calculated according to the following equation:

$$\text{Swelling index}\left(S_{w}\right)=\frac{W_{t}\;-\;W_{o}}{W_{o}}$$

where *W*_o_ is the initial weight of the dry tablet and *W*_t_ is the weight of the swollen tablet at time *t*. Data are presented as the mean±standard deviation (SD) from three samples per formulation.

**(3) Determination of floating**. The floating ability (float or sink) was determined by visual observation. The floating time was defined as the time when the tablet floated on the top surface of 500 mL of the medium (DIW) at 37.0 ± 0.5°C.

**(4) Photographic studies**. After recording the weight of the swollen tablets, tablets were then photographed using a digital camera (Canon IXUS 400, Tokyo, Japan) at the same predetermined time intervals (0.5, 2, 4, and 6 h). A swollen tablet’s characterization included information on its thickness and appearance.

### Drug solubility analysis

Solubilities of three model drugs (metformin, ciprofloxacin, and esomeprazole) were tested in media with different NaCl concentrations (0%, 0.1%, 0.5%, and 0.9%) in triplicate. Drugs were continually added to microtubes filled with 1 mL of different media to obtain saturated solutions for 72 h at 37.0 ± 0.5°C. The saturated solutions were then centrifuged at 14000 rpm for 5 min. Subsequently, the supernatants were removed and diluted to determine their concentrations by UV absorbance (V-550, Jasco, Easton, Maryland, USA) at 233, 274, and 300 nm for metformin, ciprofloxacin, and esomeprazole, respectively. Assay methods were validated using the precision and accuracy of intraday and interday assays, which demonstrated that the assay methods were all within the acceptable ranges.

### In vitro release studies

Compositions of the fabricated hydrocolloid tablets containing different model drugs (metformin, ciprofloxacin, and esomeprazole) are listed in Table 2. Dissolution tests were conducted in triplicate for all formulations by the apparatus II method (USP XXIX) (VK7020, Vankel, UK). All the release studies were performed at 100 rpm in 900 mL DIW, medium with 0.1%, 0.5%, and 0.9% NaCl, or simulated gastric solution (0.1N HCl) at 37.0 ± 0.5°C. Four-milliliter samples were withdrawn at predetermined intervals (0, 0.5, 1, 2, 3, 4, 8, 12, 16, and 24 h), and were refilled with the same volume of the fresh dissolution medium. The drug concentration was determined by the absorption of the withdrawn samples, measured spectrophotometrically (V-550, Jasco) at 233 nm for metformin, 274 nm for ciprofloxacin, and 300 nm for esomeprazole.

**Table 2:** Composition (mg) of the optimized hydrocolloid tablets containing different model drugs.

Batch code	HEC 250HHX	NaCMC 450 cps	Metformin	Ciprofloxacin	Esomeprazole
MF1–4	96	64	16	-	-
MF1–1	144	16	16	-	-
CF1–4	96	64	-	16	-
CF1–1	144	16	-	16	-
EF1–4	96	64	-	-	16
EF1–1	144	16	-	-	16

### Statistical analysis

All results are presented as the mean±SD. A one-way analysis of variance (ANOVA) was used to determine statistical significance (PASW Statistics 18.0), and *p* < 0.05 was considered statistically significant.

## Results and Discussion

### Investigation of swelling and floating properties

**Fig 1:**
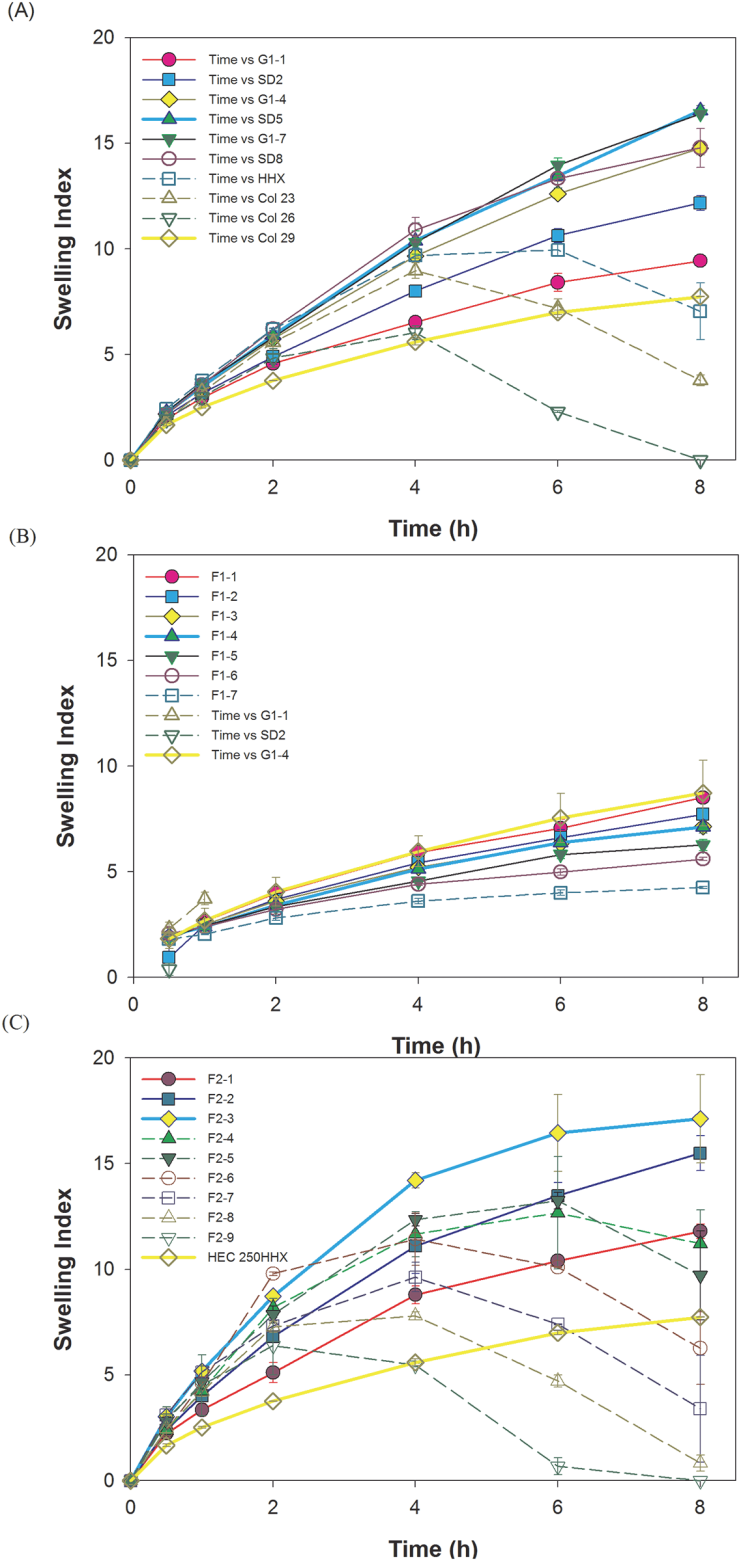
Swelling indexes of the combination of HEC 250HHX and NaCMC of 450 cps in deionized water (A) or in simulated gastric solution (B). Swelling indexes of the combination of HEC 250HHX and NaCMC of 2500 cps in deionized water (C). Representative photographs of the swollen tablets F1–1 and F1–4, after swelling under static conditions in deionized water (D).

NaCMC with different viscosities (450 and 2500 cps) was incorporated with HEC 250HHX in different ratios as shown in Table 1 as F1–0~F1–9, and F2–1~F2–9, respectively. Results demonstrate that the swelling index in DIW increased with the increasing content of the lower viscosity grade of NaCMC (450 cps) when 10–60% of NaCMC was used (Fig. 1A). The addition of NaCMC increased the swelling index in DIW due to repulsive forces of the negative charge of carboxylate carried by NaCMC (-COO^–^Na^+^). However, when the fraction of HEC was less than 50%, the cohesive force were insufficient to counteract the repulsive force presented by an amount of NaCMC greater than 40%, leading to the formation of a fluffy and crumbly gel body. Therefore, GRDDS tablets composed of 40% NaCMC of 450 cps swelled to an extremely larger size and had a very consistent texture. On the other hand, the swelling index increased with the increasing content NaCMC 450 cps was not obvious in simulated gastric solution (Fig. 1B). When the GRDDS tablets were tested in simulated gastric solution, the degree of swelling at 6 h was decreased for each formulation that contained NaCMC (compare F1–1 through F1–8 results with F1–1* through F1–8* at 6 h in Table 1)(*p*<0.05). The tablet consisting solely of NaCMC eroded in simulated gastric solution whereas it remained intact in DIW. Although F1–8 showed the highest swelling index in the beginning, the property of erosion cannot main the gel structure and then broke down within 2 h. Of note, floating duration was enhanced for formulations F1–0 through F1–8 when in simulated gastric solution compared to DIW. However formulations with 90 or 100% NaCMC 450 cps quickly eroded in the gastric simulation media.

Similarly, a large and clear gel body was observed after incorporation of the higher viscosity grade of NaCMC (2500 cps) and HEC 250HHX. The swelling index also increased when 10–40% of NaCMC of 2500 cps was used (Fig. 1C) for the same reason as for NaCMC of 450 cps. However, a weaker gel and brittle texture formed with the combination of HEC 250HHX and NaCMC of 2500 cps, and many gas bubbles trapped in the swelling zone were observed. Probably due to its much higher viscosity, air in the pore cavity of the tablet might have been trapped during tablet hydration to form gas bubbles within the gel matrix of HEC. Those trapped gas bubbles so formed produced a loose and weak structure, which might explain why the gel texture formed by NaCMC of 2500 cps was very fluffy and fragile. For the formulation of HEC and NaCMC 450 cps (F1–0 through F1–8), swelling decreased while floating duration was enhanced when in simulated gastric solution compared to DIW. The integrity of the tablets of HEC and NaCMC 2500 cps may be better maintained when exposed to acidic media. However, we assumed swelling ability is superior to floating duration, because once the tablet swells to a size that is too large to pass through the pylorus and the swelling sustains for a period of time, it results in prolonged gastric retention, while floating is often affected by the amount of stomach fluid and food uptake, so swelling ability of tablets of HEC and NaCMC 2500 cps in simulated gastric solution was not conducted.

Table 1 shows that the floating abilities of HEC hydrocolloid tablets were maintained with the addition of 10~40% NaCMC of 450 cps, whereas either sinking or temporary floating was observed for these formulations with larger amounts of NaCMC of 450 cps. In contrast, incorporation of the high-viscosity grade NaCMC (2500 cps) has a poor floating ability. In conclusion, incorporation of the low-viscosity grade NaCMC (450 cps) in proper proportions (10%~40%) in the matrix of HEC 250 HHX displayed the ability to swell without sacrificing floating.

**Fig 2:**
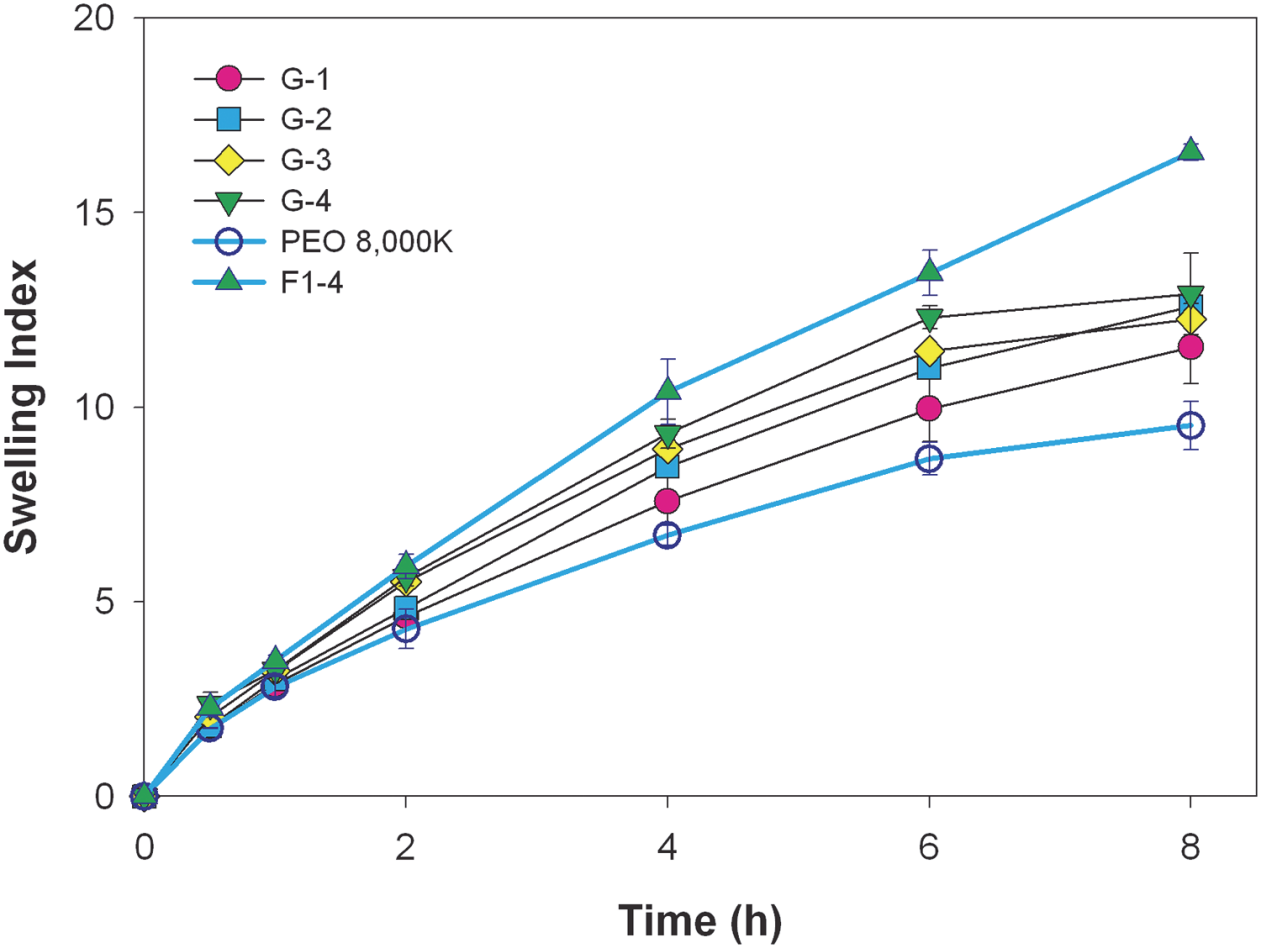
Swelling indexes of the combination of PEO 8000K and NaCMC with different ratios in deionized water .

To confirm that the swelling ability was related to the repulsive effect of negative charges of carboxylate groups carried by NaCMC, up to 40% of NaCMC of 450 cps was incorporated with another neutral hydrophilic polymer of PEO 8000K, as shown in Table 1 (G-0~G-4). Similar results were obtained; for example, the swelling index soared when the amount of NaCMC increased (Fig. 2). However, the swelling size obtained from F1–4 formulation was larger than those of PEO formulations. This reinforced the notion that incorporation of swellable ionic polymers of an appropriate viscosity, such as NaCMC of 450 cps, synergistically promoted the swelling ability. Unlike incorporation of the HEC 250HHX matrix, sinking was observed with the PEO 8000K formulations regardless of the amount of NaCMC of 450 cps added, as demonstrated by the results listed in Table 1.

**Fig 3:**
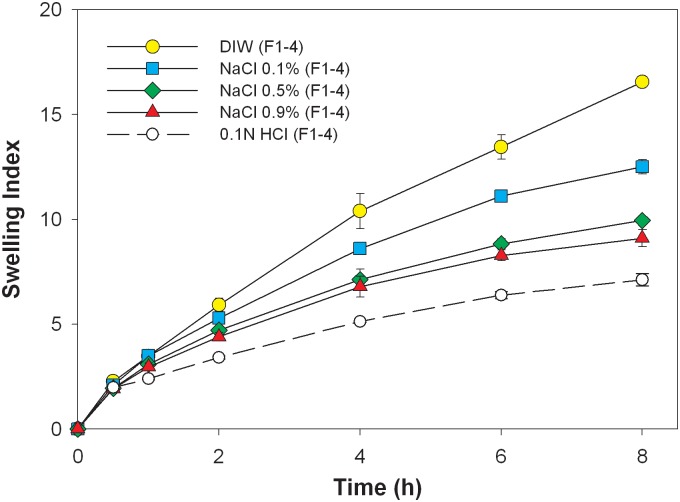
Swelling indexes of F1–4 in mediums with different concentration of NaCl .

Formulation F1–4 was further examined in media with different NaCl concentrations (0%, 0.1%, 0.5%, and 0.9%), and the swelling index versus time is shown in Fig. 3. The figure shows that the swelling index decreased when the concentration of NaCl in the medium increased (*p*<0.05 compared to DIW at 8 h). This can possibly be attributed to a shielding effect of electrolytes to the ionized carboxylic group (-COO^-^) of NaCMC, and it could make expansion of the hydrated matrix by repulsive forces less effective. As expected, the influence of the repulsive force expressed by NaCMC on the swelling index was less with a decreasing NaCMC content in the matrix of HEC tablets.

**Fig 4:**
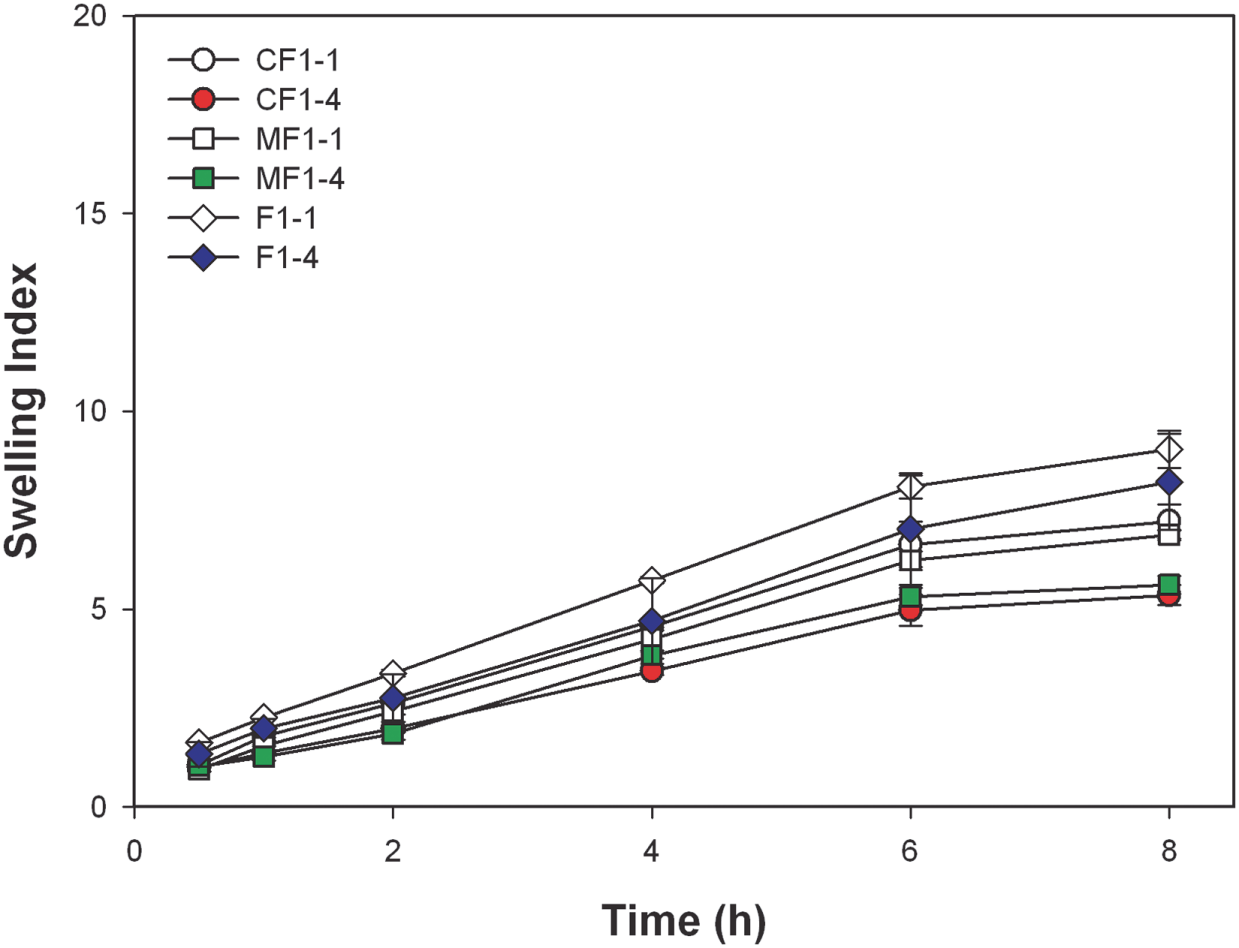
Swelling indexes of F1–1, F1–4, and F1–1 and F1–4 incorporated with metformin and ciprofloxacin in simulated gastric solution .

Fig. 4. shows the swelling index of F1–1 and F1–4 when incorporated with metformin and ciprofloxacin in simulated gastric solution, excluding esomeprazole because of its instability in acidic conditions. The addition of either metformin or ciprofloxacin decreased the swelling index regardless of F1–1 or F1–4. However, no matter in F1–1 or F1–4, there was insignificant difference between metformin and ciprofloxacin (*p* = 0.27 and *p* = 0.25 for F1–1 and F1–4 at 8 h, respectively).

### Drug solubility analysis

**Fig 5:**
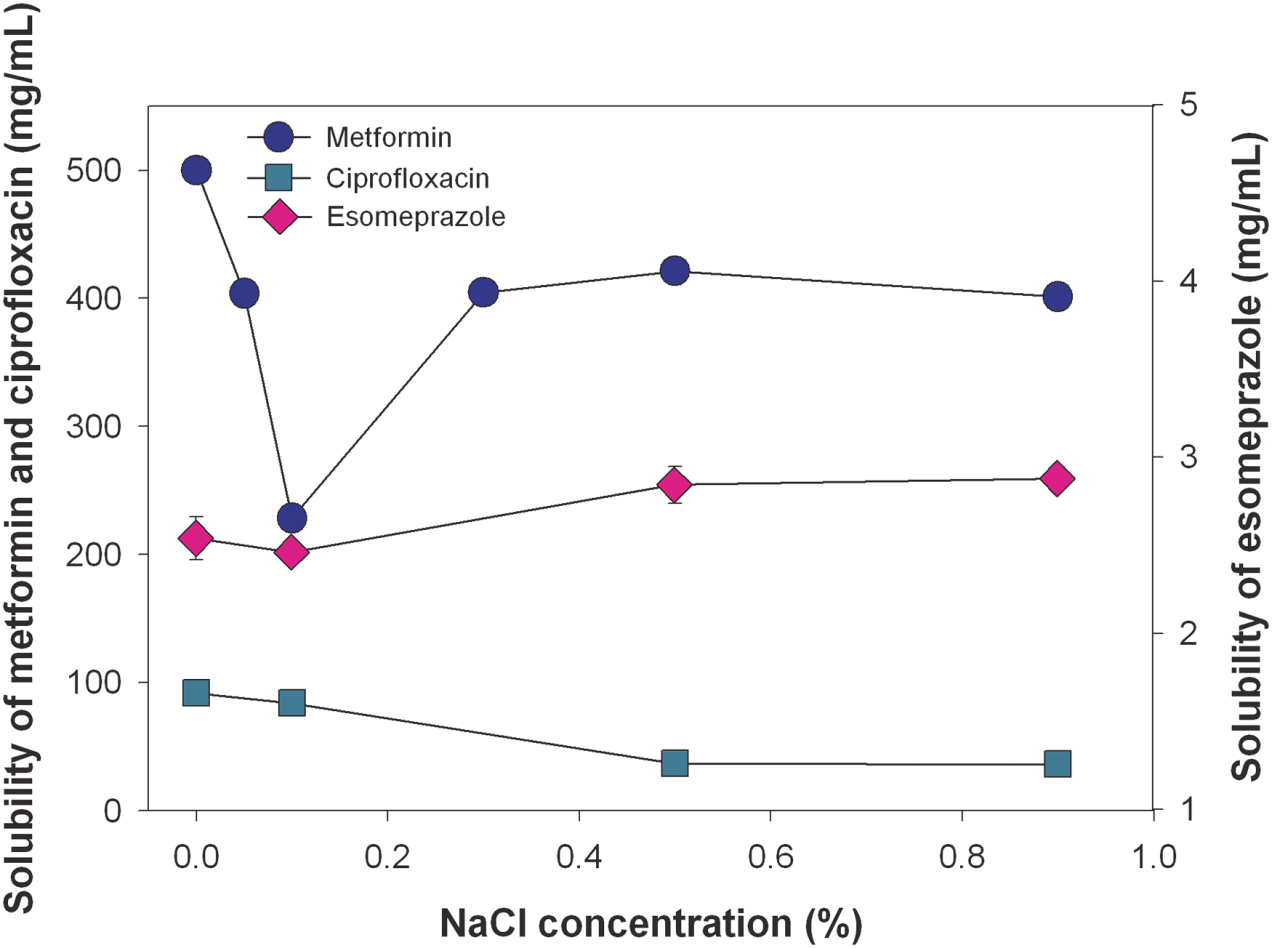
Solubilities of the three model drugs (metformin, ciprofloxacin, and esomeprazole) in mediums with different concentration of NaCl .

Since metformin and ciprofloxacin are supplied in an HCl salt form and esomeprazole is in a Na salt form, their solubilities were expected to be influenced by the NaCl concentration in the medium mainly due to the common ion effect and slightly due to an ionic strength effect. Results of the solubility analysis Fig. 5 show that the solubilities among the three model drugs were in the rank order of metformin > ciprofloxacin >> esomeprazole. The solubilities of freely water-soluble metformin was within 401 to 500 mg/mL in 0%, 0.05%, 0.3%, 0.5%, and 0.9% NaCl media. However as revealed, the solubility of metformin dropped to 228 mg/mL in a 0.1% NaCl concentration medium, and then rose with increasing NaCl concentrations. The drug solubility of water-soluble ciprofloxacin was within the range of 36 to 91 mg/mL, in which the solubility decreased with increasing NaCl concentrations, leading to the solubility of as low as 36 mg/mL in 0.9% NaCl medium. For the slightly water-soluble drug esomeprazole, drug solubility was 2.46 to 2.88 mg/mL, and its solubility slightly increased at higher NaCl concentrations.

### The drug release studies

**Fig 6:**
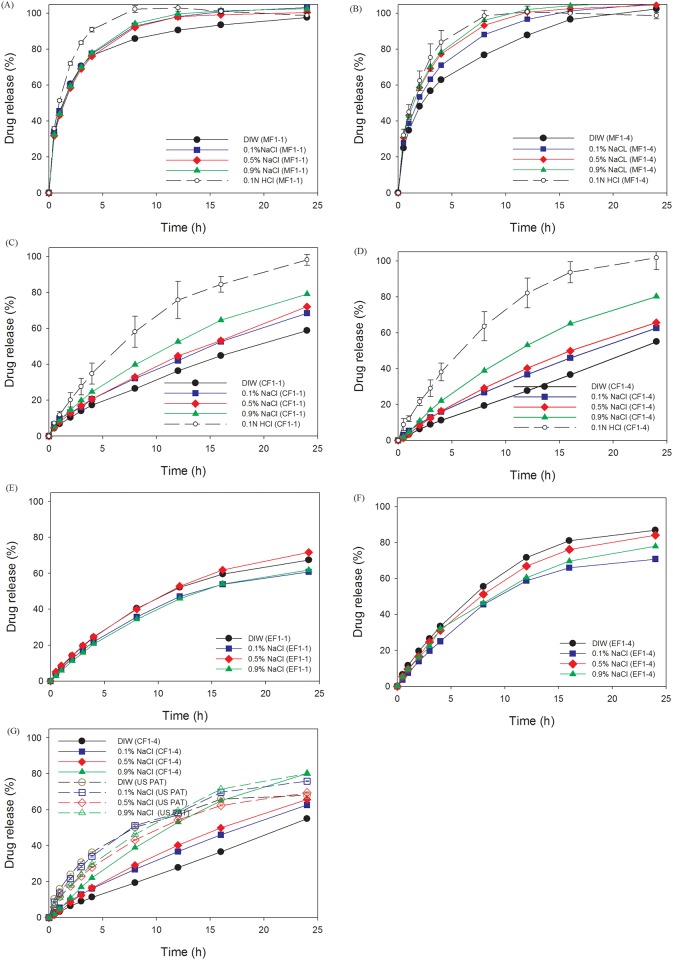
In vitro release profiles of metformin (A and B), ciprofloxacin (C and D), and esomeprazole (E and F) in simulated gastric solution and mediums with different concentrations of NaCl, and comparison of U.S. patent example and CF1–4 of ciprofloxacin in mediums with different concentrations of NaCl (G).

Formulation F1–4 and F1–1 had the greatest extent of swelling with a floating ability in DIW and simulated gastric solution were selected for incorporation of three model drugs with different solubilities (metformin (M), ciprofloxacin (C), and esomeprazole (E)). Drug-release patterns in media with different NaCl concentrations for the three model drugs incorporated in the formulations F1–4 and F1–1 were investigated, and the results are shown in Fig. 6. MF1–1 was completely released in less than 10 h; meanwhile, release rates ascended with increasing NaCl concentrations, which were opposite to the swelling rates.

According to the model for Case II, which was based on the work of Alfrey on swelling [37], drugs in the swollen matrix were completely released and drugs in the swelling zone were ready for release when the matrix was completely swollen; hence, the moving rate of the swelling zone was the determination factor of the drug-release rate. This system was titled “Case II Diffusion.” In these swollen tablets, the swelling zone additionally determined the moving rate of the polymer matrix. Therefore, if the surface area of the tablet could remain consistent (constant), then the drug-release rate would be constant. The drug-release percentage (*M*_*s*_/*M*_∞_ × 100) from the swelling matrix versus time (*t*) was then be expressed by the equation below (the power law), where *n* is the release exponent, and *k* is the release-rate constant:

$$M_{s}/M_{\infty }\times 100=kt^{\text{n}}$$

If the release mechanism was dependent on diffusion from the cylindrical tablet’s swelling matrix, then the *n* would be 0.45. If the release mechanism was based on the swelling zone’s moving rate in a cylindrical device, then *n* would be 0.9. Therefore, when the *n* value was between 0.45 and 0.9, the release mechanism was affected by both mechanisms.

A log-log plot demonstrates the compliance with the relationship of the power law (all *r* ^2^ values given in Table 3 were > 0.99). Furthermore, the parameters of the release-rate constant (*k*) and the calculated release exponent (*n*) are also tabulated in Table 3. Results show that the release rate of metformin rose in media with NaCl concentrations of 0.0% and 0.5%, and were similar in the media containing 0.5% and 0.9% of NaCl (the release-rate constant, *k* value, was in the order 1.54 < 1.59 < 1.63 ≈ 1.63, respectively). Metformin is a freely water-soluble drug; therefore, the rate-determining step in the drug release was the swelling rate of the matrix. Therefore, the length and resistance of the diffusion paths determined the release profiles of metformin in media containing different NaCl amounts. As described in section 3.1, the swelling index and gelled layer of F1–4 tablets decreased when NaCl increased in the medium, leading to an increasing diffusion rate. The gel layer was thicker in DIW than that in NaCl media. Thus, the release rate of metformin was slower in DIW and soared with increasing NaCl concentrations in the media. However, this inhibitory effect was insignificant when lower amounts of NaCMC were incorporated in the MF1–1 formulation, resulting in similar release rates in media containing different levels of NaCl. Conversely, the release exponent *n* = 0.45 (for cylindrical tablets) indicates that Fickian diffusion was dominant in the dissolution of both MF1–4 and MF1–1. Good correlations are shown in Table 3. However, the expected decrease in the release rate constant in 0.1% NaCl medium was not found, although the solubility of metformin in this medium was the lowest among those examined. One explanation for this might be that the solubility of metformin in 0.1% NaCl was still higher than 200 mg/mL, which was not low enough to have a significant effect on the release rate of metformin in 0.1% NaCl medium.

**Table 3:** Parameters of the release rate constant (*k*), release exponent (*n*), and correlation coefficient (*r* ^2^) based on the power law for the release of metformin, ciprofloxacin, and esomeprazole from formulations F1–1 and F1–4.

	NaCl Concn. (%)	Release rate constant (*k*)	Release exponent (*n*)	Correlation coefficient (*r* ^2^)
Metformin (MF1–4)	0.0	1.54	0.45	0.9979
	0.1	1.59	0.45	0.9993
	0.5	1.63	0.44	0.9982
	0.9	1.63	0.44	0.9993
	0.1N HCl	1.65	0.47	0.9991
Metformin (MF1–1)	0.0	1.65	0.38	0.9980
	0.1	1.66	0.40	0.9987
	0.5	1.62	0.43	0.9984
	0.9	1.64	0.42	0.9994
	0.1N HCl	1.70	0.45	0.9924
Ciprofloxacin (CF1–4)	0.0	0.46	0.94	0.9896
	0.1	0.73	0.78	0.9997
	0.5	0.54	1.02	0.9825
	0.9	0.66	1.02	0.9841
	0.1NHCl	1.08	0.81	0.9991
Ciprofloxacin (CF1–1)	0.0	0.83	0.67	0.9993
	0.1	0.96	0.61	0.9919
	0.5	0.89	0.70	0.9998
	0.9	0.94	0.73	0.9995
	0.1N HCl	1.07	0.76	0.9998
Esomeprazole (EF1–4)	0.0	1.06	0.77	0.9996
	0.1	0.85	0.91	0.9983
	0.5	0.98	0.82	0.9986
	0.9	0.97	0.80	0.9938
Esomeprazole (EF1–1)	0.0	0.87	0.85	0.9958
	0.1	0.84	0.82	0.9972
	0.5	0.94	0.74	0.9997
	0.9	0.75	0.92	0.9948

The release rate of ciprofloxacin incorporated in CF1–4 and CF1–1 decreased within 24 h in media containing NaCl of 0.9%>0.5%>0.1%>0.0% for both formulations, and a slightly higher release rate was obtained in CF1–1 than CF1–4 (Fig. 6C, D), the extent of influence of which decreased with increasing NaCl concentrations in the media. As shown in Table 3, release rates (*k* value) of ciprofloxacin from CF1–4 and CF1–1 in media with increasing concentrations of NaCl were 0.46, 0.73, 0.54, 0.66, and 0.83, 0.96, 0.89, and 0.94, respectively, which indicates that overall release rates were faster in CF1–1 compared to those in CF1–4. This might have been caused by the acid-base interactions between the carboxyl groups of NaCMC and the aliphatic piperazine nitrogen of ciprofloxacin, which also was shown by Bermúdez et al. [38]. The extent of acid-base interactions was unapparent with a lower amount of NaCMC, so the release rate increased when ciprofloxacin was incorporated in CF1–1, whereas such an interaction was hindered by the increased ionic strength, resulting in a faster release rate in the higher NaCl medium.

Tablets made from CF1–4 and CF1–1 slightly swelled into a larger size without the formation of an obviously thick gel layer. It seemed that an interaction existed between ciprofloxacin and NaCMC, as described, resulting in the hindrance of the swellability of NaCMC in the HEC matrix. A strong correlation was found as shown in Table 3 as well; the release exponents *n* = 0.78~1.02 from CF1–4 and *n* = 0.61~0.73 from CF1–1 indicate that the release mechanisms of both formulations were by anomalous diffusion, in which relaxation-controlled release was dominant in CF1–4, and diffusion-controlled release was dominant in CF1–1. Apparently, the greater extent of acid-base interactions in CF1–4 than in CF1–1 contributed to the modulation of the release mechanism.

For the slightly water-soluble drug, esomeprazole, incorporated in EF1–4 and EF1–1, the rank order of the decreasing release rates in the medium containing various NaCl concentrations was 0.0%>0.5%>0.9%≒0.1% and 0.0%≒0.5%>0.9%≒0.1%, respectively, with faster release rates in EF1–4 than in EF1–1 in the same media (Fig. 6E, F). As shown in Table 3, release rates (*k* value) of esomeprazole from EF1–4 and EF1–1 in media with increasing NaCl concentrations were 1.06, 0.85, 0.98, 0.97 and 0.87, 0.84, 0.94, and 0.75, respectively. With a higher content of NaCMC in EF1–4, the influence of the ionic strength on gel formation by NaCMC was greater, leading to the formation of a fluffy and crumbly gel layer that caused a faster release rate in EF1–4. This explains why the release rate of esomeprazole from EF1–4 was faster than that from EF1–1 in the same medium. Solubilities of esomeprazole in media with different NaCl concentrations were in the order of 0.0%≒0.1%<0.5%<0.9%. However, there was no significant difference, so the impact of solubility on the release rate was inconclusive. The release exponents *n* = 0.76~0.91 for EF1–4 and *n* = 0.74~0.91 for EF1–1 was close to *n* = 0.9, indicating that the underlying mechanism governing the drug release of esomeprazole from both formulations was approximately the moving rate of the swelling zone.

In the example shown in US Patent 6723340 [21], the components, including PEO 8000K, Metolose 60-SH of 4000 cps, and microcrystalline cellulose 101, were mixed with ciprofloxacin, and were regarded as the reference group. The release rate of the reference group was faster compared to CF1–4 except in a 0.9% NaCl medium (Fig. 6G). The obtained results demonstrated that the retardation of the release rate was more noticeable in our developed formulations than that in the patented formulations.

**Fig 7:**
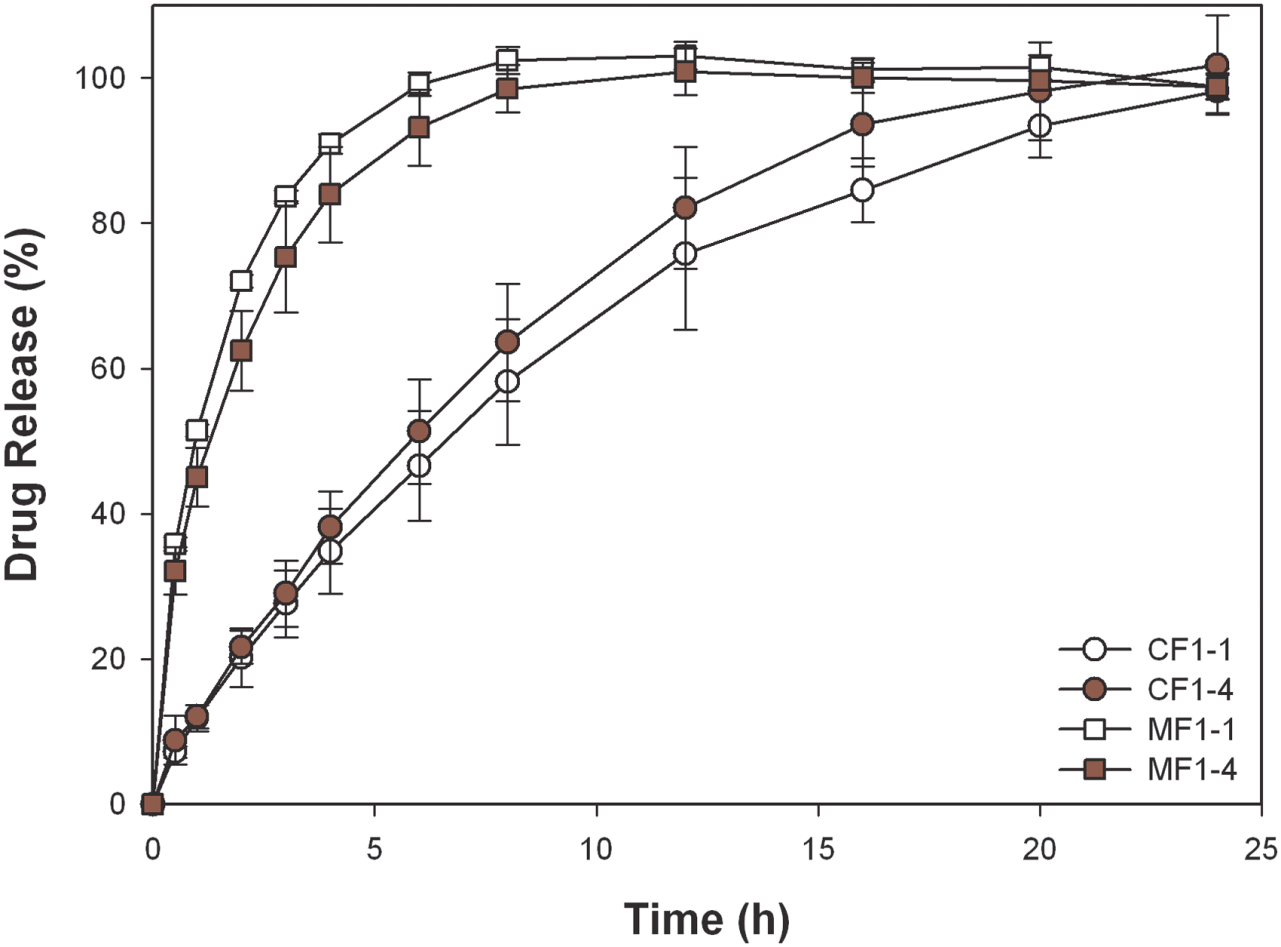
In vitro release profiles of metformin and ciprofloxacin in simulated gastric solution.

Results of the *in vitro* release and swelling property of metformin and ciprofloxacin in simulated gastric solution from two formulations (F1–1 and F1–4) are plotted in Fig. 7 and 4 for comparison, excluding esomeprazole because of its instability in acidic conditions. The higher solubility of metformin may contribute to the faster release rate in simulated gastric solution compared to that of ciprofloxacin. One of the rate-determining steps in drug release was the swelling rate of the matrix. Therefore, the length and resistance of the diffusion paths determined the release profiles in media. The swelling index of CF1–4 tablets was lower than that of CF1–1 in acidic medium, leading to a faster diffusion and release rate. However, the relationship of swelling index and drug release rate for metformin was less apparent probably because the freely water-soluble property is superior to the swelling ability in determining release. The characteristic parameters of the power law listed in Table 3 show that the release mechanism (release component *n* value) from two formulations was maintained, but the release rate (release rate constant *k*) was slightly accelerated. The acceleration of the release rate seemed to have been more profound for ciprofloxacin from formulation CF1–4. A possible explanation for the phenomena might be a slight retardation of the acidic pH value on gel formation of NaCMC, causing a less-viscous pathway for drug diffusion with no influence on the release mechanism. As a result, by increasing the fraction of NaCMC in the formulation, such as MF1–4 versus MF1–1, or CF1–4 versus CF1–1, the extent of retardation was increased, leading to a more significant increase in the release rate.

## Conclusion

The findings of this study showed that in DIW the combination of HEC 250HHX and NaCMC of 450 cps in a ratio of 60:40 exhibited an enhanced swelling index (13.45) with no deterioration of the floating period due to repulsive forces of the negative charge carried by NaCMC, which was substantially better than the combinations of NaCMC of 2500 cps with PEO 8000K. When the formulation contained HEC 250HHX and NaCMC 450 cps tested in simulated gastric solution, the degree of swelling was decreased, while floating duration was enhanced. Dissolution profiles of these three model drugs (metformin, ciprofloxacin, and esomeprazole) with different solubilities from the optimized GRDDS of F1–4 were also demonstrated to follow a sustained pattern in aqueous solutions and simulated gastric media. Due to the decreased swelling ability and hence less resistance of the diffusion in simulated gastric solution, the release rate of metformin and ciprofloxacin was faster than those in DIW. Overall, the developed swellable/floatable GRDDS composed of HEC 250HHX and NaCMC of 450 cps are expected to potentially be retained in the stomach and to release drugs in a sustained manner, which would be beneficial for those drugs that are only absorbed in the stomach or in the upper small intestine, exhibit local efficacy in the stomach, are unstable, or have low solubilities in high-pH environments.
